# HIV-1 envelope glycoproteins isolated from Viremic Non-Progressor individuals are fully functional and cytopathic

**DOI:** 10.1038/s41598-019-42075-3

**Published:** 2019-04-03

**Authors:** Romina Cabrera-Rodríguez, Veronique Hebmann, Silvia Marfil, María Pernas, Sara Marrero-Hernández, Cecilia Cabrera, Victor Urrea, Concepción Casado, Isabel Olivares, Daniel Márquez-Arce, Silvia Pérez-Yanes, Judith Estévez-Herrera, Bonaventura Clotet, Lucile Espert, Cecilio López-Galíndez, Martine Biard-Piechaczyk, Agustín Valenzuela-Fernández, Julià Blanco

**Affiliations:** 1 Laboratorio de Inmunología Celular y Viral, Unidad Virología y Microbiología del IUETSPC, Unidad de Farmacología, Sección de Medicina, Facultad de Medicina, Universidad de La Laguna (ULL), La Laguna, 38071 Tenerife Spain; 2 Institut de Recherche en Infectologie de Montpellier (IRIM), Université de Montpellier, CNRS, 34293 Montpellier, France; 3 AIDS Research Institute IrsiCaixa, Institut de Recerca en Ciències de la Salut Germans Trias i Pujol (IGTP), 08916 Badalona, Barcelona, Catalonia Spain; 4 Unidad de Virología Molecular. LRIR. Centro Nacional de Microbiología (CNM), Instituto de Salud Carlos III, 28220 Majadahonda, Madrid, Spain; 5 Universitat de Vic-Central de Catalunya, UVIC-UCC, Vic, 08500 Catalonia Spain

## Abstract

In untreated HIV-1-infected individuals, viremia is positively associated with disease progression. However, some viremic non progressors (VNPs) individuals show paradoxical high CD4^+^ T cell counts. HIV-1 envelope glycoprotein complex (Env) is a major cytopathic determinant in viral replication; therefore, we have deeply characterized Env function in this rare clinical phenotype. Full-length Env clones isolated from individuals with Viral Load (VL) > 10,000 copies/mL classified as VNPs (n = 15) or rapid progressors (RPs, n = 17) were geno- and phenotypically analyzed by determining diversity, expression, CD4 binding/signaling, fusogenicity, infectivity and autophagy induction. Selected Env clones from VNPs and RPs (n = 32) showed similar expression, fusion and infection abilities. Env clones from both groups showed similar affinity for CD4 during cell-to-cell transmission and consistently induced similar levels of CD4 signaling, measured by α-tubulin acetylation. Moreover, we demonstrate for the first time that primary Env clones from VNP and RP induce autophagy in uninfected cells and that this feature correlated with fusogenic capacity but was unrelated to disease progression. In conclusion, our data suggest that Env clones from VNP individuals are fully functional. Therefore, the paradoxical CD4^+^ T cell count stability coexisting with high levels of viral replication is unrelated to Env function.

## Introduction

Human immunodeficiency virus type 1 (HIV-1) infection destroys CD4^+^ T cells and compromises the function of the immune system leading to acquired immunodeficiency syndrome (AIDS)^1,2^. However, the rate of CD4^+^ T-cell depletion and the time to onset of AIDS symptoms are highly variable among HIV-1 infected individuals^3^. This variability defines several clinically relevant groups of HIV-1 infected individuals, such as long-term non progressors (LTNPs) that show, in general, a low viremia level (below standard detection limits in some cases) and a slow progression to AIDS^3^. The reduced level of viral replication has been associated to the magnitude and quality of the immune responses, in particular CD8-mediated control, which results in low or even suppressed viral replication^4^. Virological factors that impair the *in vivo* viral fitness have also been shown to contribute to this phenotype^5^. In an opposed setting, high levels of viral replication, either as a consequence of poor or inefficient immunological responses or particular viral cytopathic factors, are associated with rapid progression to AIDS^6–8^.

Besides these well-characterized clinical phenotypes, in an extremely low percentage of patients, known as viremic non-progressors (VNPs), a high level of viral replication is accompanied by a paradoxical slow CD4^+^ T-cell destruction^9^. Little is known about the reasons that may explain the apparent non-cytopathic viral replication. However, it is reasonable to speculate that both immunological and virological factors are at play^10^. From an immunological point of view, VNPs do not show enhanced cytotoxic T lymphocyte (CTL) responses^11^ although they seem to control exacerbated type I-interferon-mediated responses present in HIV-1 infected individuals^10^. This setting could be reminiscent of the non-progressive SIV infection described in sooty mangabeys and might maintain a relatively protected CD4 central memory subset, a key population of the CD4^+^ T-cell compartment^12,13^. From a virological point of view the available information is quite limited. We have previously described the isolation of full-length HIV-1 envelope genes (*env*) from a small group of VNPs^9^. This study showed that the viral tropism was R5 and that these *env* clones were functional regarding fusogenicity and ability to induce the expression of NKp44L on CD4^+^ T cells^9^. Additional available information suggest that viral replication capacity of viruses isolated from VNPs is impaired, whereas a maintained Nef functions has been described^14,15^.

Assuming the widely described major role of Env in viral fitness and pathogenesis^5,16–18^, we hypothesized that Env isolated from VNPs might have specific features leading to the VNP clinical outcome. To test this hypothesis, we have deeply characterized full-length Env clones isolated from VNPs by evaluating their genotypical and phenotypical features (CD4 binding, signaling capacity and autophagy induction). All these features were compared to Env isolated from RPs. Our data show that VNPs harbor fully signaling-and fusion-competent Envs, which also show fully cytopathic potential as assessed by their ability to induce autophagy in bystander uninfected CD4^+^ T cells.

## Results

### Samples and Env clones

We have previously isolated a large collection of full-length Env clones from four VNP and five RP individuals. Table 1 shows the main features of selected individuals. All VNP individuals showed plasma HIV-1 VL > 10,000 copies/mL with relatively high (>400 cells/µL) and stable levels of CD4^+^ T cells^9^. Patient 8 received antiretroviral therapy from 1997 to 2002, and after 2004. Plasma samples selected for this study belong to the period when the patient was off therapy. In contrast, individuals identified as RPs showed lower CD4^+^ T cell counts (median 358 cells/µL) with HIV-1 VL comparable to VNPs (median 95,960 copies/mL). All samples from this group were collected within 3 years after seroconversion (median 1 year, Table 1).

**Table 1:** Main features of individuals included in the study.

Group	Patient ID	Plasma VL (copies HIV-1 RNA/mL)	CD4^+^ T cell count (cells/µL)	Time from HIV-1 infection (years)
VNPs				
	VNP8	168,193	945	7
	VNP9	126,383	486	5
	VNP11	25,144	995	11
	VNP16	31,523	735	4
**Median VNPs**		**78,953**	**840**	**6**
RPs				
	RP2	226,149	180	0.8
	RP6	76,965	436	1
	RP7	95,960	358	1
	RP8	500,000	262	2.5
	RP10	53,026	359	1
**Median RPs**		**95,960**	**358**	**1**

### Characterization and selection of cloned *env*

**Figure 1:**
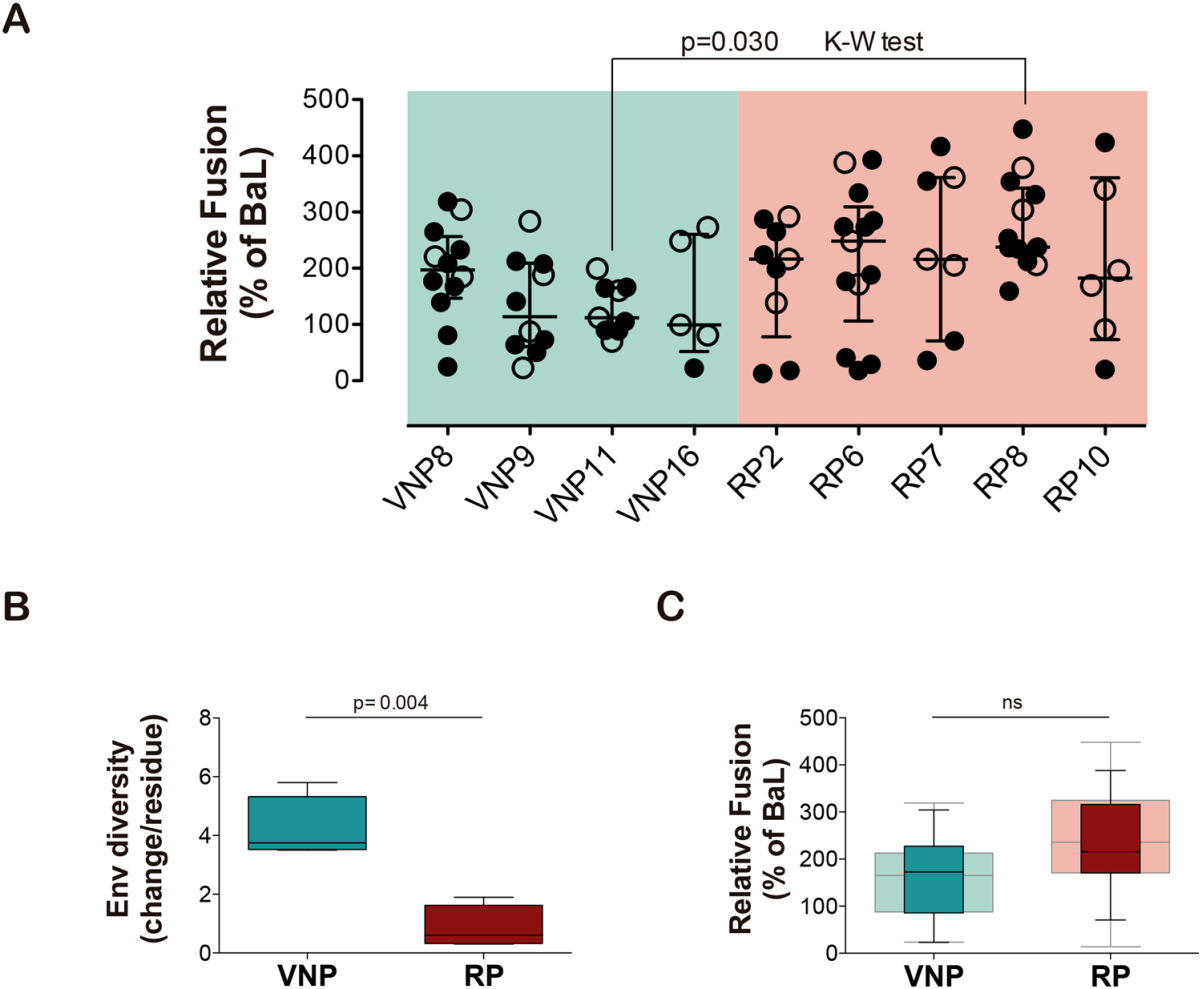
Analysis of genotypic and phenotypic features of HIV-1 envelopes isolated from VNP and RP individuals. (**A**) Relative fusion activity of the full Env collection (BaL = 100%). Individual points correspond to the mean of triplicate independent experiments. Median fusogenicity of the Envs isolated from each participant was compared (Kruskal-Wallis test). Empty symbols, both in VNP and RP groups, indicate Envs that we select in this study, from the full collection of isolated Env clones (100 full-length *env* clones, as indicated below), to be further characterized in their viral functionalities. This represents a selection of 32 Envs, 15 for VNPs and 17 for RPs. (**B**) Boxplot comparison of the mean diversity of the full gp160 sequence from all clones isolated from VNPs (n = 4, green) or RPs (n = 4, red). Comparison was performed using the Mann Whitney test. (**C**) Boxplot comparison (median and interquartile range) of relative fusion of selected *env* clones (n = 15 for VNPs and n = 17 for RPs). Statistical analysis was performed using Mann Whitney test. Data from full *env* collection (n = 36 and n = 64 for VNPs and RPs, respectively) is shown lighter on the background for comparative purposes.

In a previous work, we characterized the fusogenic activity of cell-surface expressed HIV-1 Envs isolated from plasma samples obtained from VNP and RP participants. A total of 100 clones (all showing an R5 tropism) were tested for fusogenicity in co-cultures of Tat/Env cotransfected HEK-293T cells and TZM-bl cells^9^. Figure 1A shows fusion capacity (relative to BaL) of all clones. Comparison of the median fusogenicity of Env isolated from each individual showed only significant differences between VNP11 and RP8 individuals (p = 0.03, Kruskal-Wallis test), indicating that no major difference in fusogenicity was evident among Envs isolated from VNPs and RPs at the individual level. Remarkably, most plasma isolated Envs showed higher fusogenicity than the reference BaL-Env (values > 100%).

To further characterize Env features, we genotypically analyzed the global *env* gene sequence collection, except for participant VNP10, which showed a recombinant F/B Env sequence. Consistent with longer time from seroconversion in VNPs (Table 1), the mean diversity of Env clones isolated from RPs was significantly lower than those from VNPs (p = 0.004, Mann-Whitney test, Fig. 1B). However, no significant correlation was observed between fusogenicity and diversity when all available participants were analyzed. Furthermore, for each clone, genetic distance to the patient consensus sequence was calculated; again, no correlation of genetic distances and fusogenicity was observed (data not shown).

### Phenotypic characterization of selected Env: expression and infectivity

Further phenotypic characterization of Env was performed in a selected collection of 32 Env clones (15 from VNPs and 17 from RPs, empty dots in Fig. 1A). The selected Env recapitulate the whole Env collection, showing similar median fusogenicity values and distribution (dark and light boxes for whole collection and selected Env, respectively in Fig. 1C).

**Figure 2:**
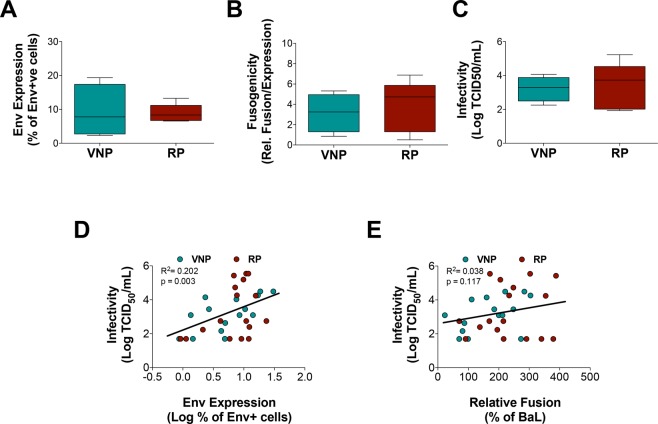
Analysis of specific fusogenicity and infectivity of selected HIV-1 envelopes isolated form VNP and RP individuals. Selected Env from VNPs (green) and RPs (red) were analyzed for expression after transfection in HEK-293T cells (panel A). The specific fusogenicity was calculated as the ratio of fusion activity and the number of Env cell-surface expressed molecules (panel B). Pseudoviruses bearing selected Env were tittered in TZM-bl cells. Titers are shown in panel C. Comparisons show mean values of selected Env from each participant (VNP n = 4, RP n = 5). No statistical differences were found (Mann Whitney test). Association of Infectivity of cell free viruses and Env expression (panel D) and fusogenicity (panel E). Green and red dots correspond to VNP and RP clones, respectively.

Selected Envs were first analyzed for cell-surface expression in transfected HEK-293T cells. Similar levels of Env expression were observed for VNP and RP groups when mean values of selected Env from each participant were compared (Fig. 2A). Consistently, the means of specific fusogenicity (defined as fusion activity normalized by Env expression) were also similar for Env isolated from VNP or RP individuals (Fig. 2B).

Since all the above-described parameters were analyzed in a setting of cell-to-cell fusion events, we next evaluated Env-mediated fusion during cell-free viral infection. The infectivity of pseudoviruses bearing different Env was measured as infectious titers in TZM-bl cells. Mean infectivity of clones from each participant showed again similar values for both groups (Fig. 2C). When Envs from VNP and RP were analyzed together and considering multiple measures from each participant, viral infectivity was positively associated with Env expression but not with specific fusogenicity (Fig. 2D,E). Since, these relationships were more strongly observed in VNP Env, we performed a multivariate analysis of linear mixed effect model with random intercepts to explore specific differences between VNP and RP Envs. This analysis showed that the only independent parameter that is associated with infectivity was the level of Env expression, while fusogenicity or clinical group (RP or VNP) had no significant impact on infectivity or fusion activity (Table 2).

**Table 2:** Analysis of factors determining viral infectivity.

Infectivity vs	Value^*a*^	Standard Error	P-value
Log_10_ (Expression)	1.3562	0.4874	0.0055
Fusogenicity	0.0016	0.0019	0.3527
Group (VNP or RP)	−0.0557	0.6683	0.9076

Data represent the slope of multivariate linear mixed effect model with random intercepts. Inference was assessed through likelihood ratio test comparing the full model with the model without one of the predictors. A selected collection of 32 Env clones (15 of VNPs and 17 of RPs), which correspond to empty points in Fig. 1A was used for the analysis.

### CD4 binding and signaling activity of selected Envs

**Figure 3:**
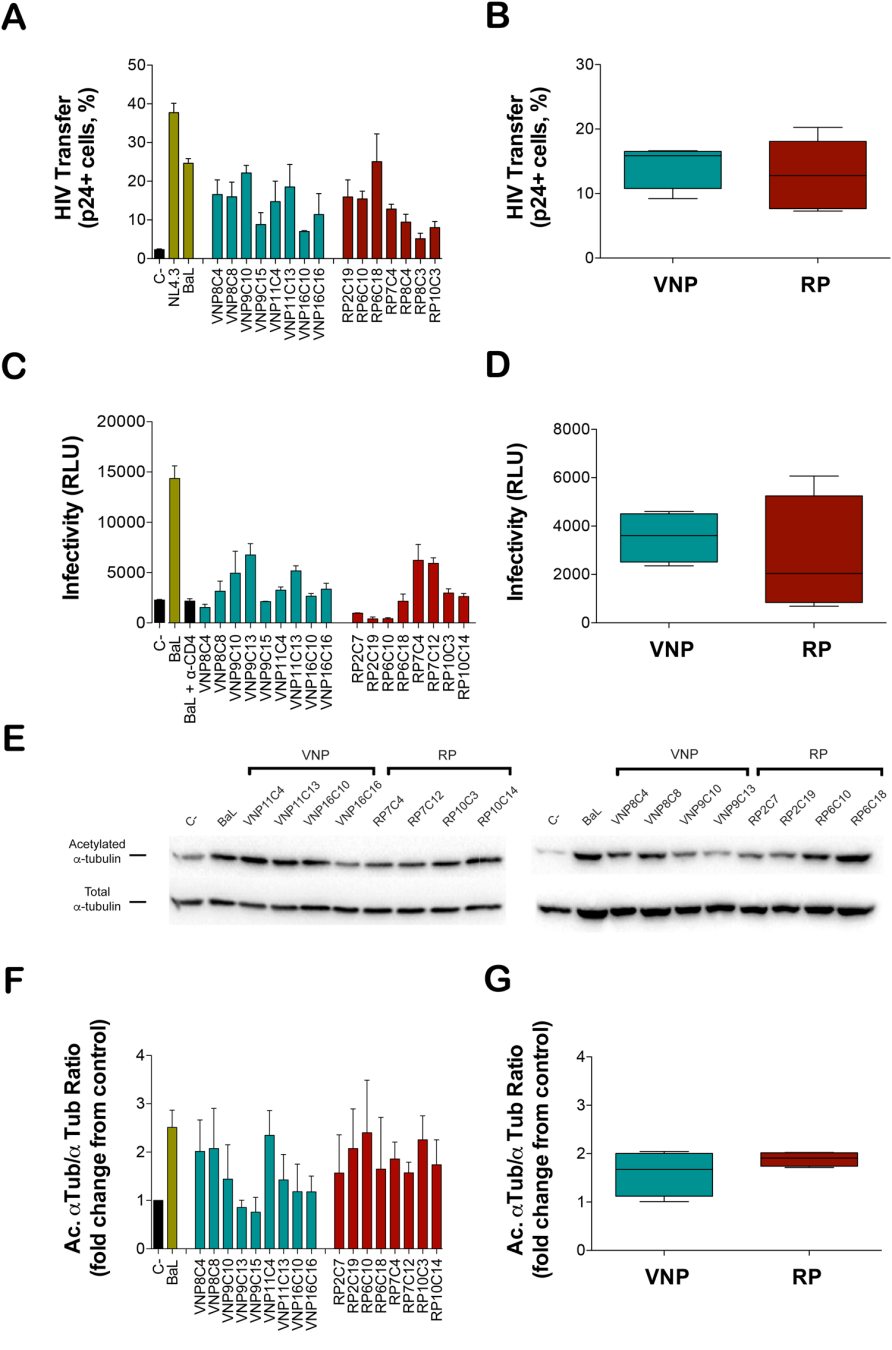
Analysis of CD4 binding efficiency and CD4 mediated signaling induced by selected HIV-1-Env isolated from VNP and RP individuals. (**A**) The interaction of Env with CD4 was evaluated in co-cultures of HEK-293T producing pseudoviruses bearing the indicated Env clones with primary CD4^+^ T cells. The figure shows Virus binding to CD4 as the % of p24+ cells assessed by flow cytometry. Panel A shows mean ± SEM of four different experiments for the indicated clones. Panel B shows the comparison of mean values of virus transfer for VNP and RP individuals. Panels C and D show Luciferase-based assay of viral infection in permissive CEM.NKR-CCR5 cells by non-replicative HIV-1 luciferase reporter pseudoviruses bearing Envs isolated form VNPs (green bars) and RPs (red bars), and control BaL.01-Env strain (gray bar). Non-productive infection values (baseline) are obtained with a neutralizing anti-CD4 mAb (5 μg/mL), under the same experimental conditions. Data in panel C are mean ± SEM values of nine independent experiments carried out in triplicate, while panel D show global comparison of mean values of virus transfer for VNP and RP individuals. Panel E shows a representative quantitative western-blot analysis of Envs-induced α-tubulin acetylation during early infection in permissive CEM.NKR-CCR5 cells comparing VNP and RP Env. Data are represented as acetylated α-tubulin in reference to total α-tubulin (Ac. α-tub/α-tub ratios). These western-blot bands are key bands from full-length gels and blots presented in Supplementary Fig. 1. First, in each gel/blot, post-transductional acetylation of α-tubulin is detected, then membranes were gently stripped and blotted again to detect total α-tubulin in the same gel/blot, as described in Methods section. Therefore, acetylated α-tubulin and total α-tubulin and detected in the same gel/blot, under any experimental condition. Panel F shows histograms of the quantification of HIV-1 Env-mediated α-tubulin acetylation in permissive cells from three independent experiments carried out in triplicate, per each experiment condition. Data are mean ± SEM.; Ac. α-tub/α-tub ratios. Panel G shows comparison of mean values of virus transfer for VNP and RP individuals.

To further dissect potential differences among Envs isolated from VNP and RP individuals, we analyzed the Env attachment to CD4, by determining both the binding efficiency and the intracellular signals delivered into target cells. First, we assessed gp120/CD4 binding efficiency by taking advantage of the highly efficient virus binding to CD4 that occurs during virological synapses^19,20^. Indeed, early HIV transfer between infected and uninfected cells is independent of coreceptor expression or fusion activity and is strictly dependent on the binding of gp120 to CD4^5^. HEK-293T cells producing viral particles pseudotyped with Env isolated from VNP or RP individuals were co-cultured with primary human CD4^+^ T cells and the amounts of viruses captured by target cells was analyzed by flow cytometry^19,20^. In contrast to fusogenicity, CD4 binding capacity of primary Env was similar or lower than the reference isolates BaL- or NL4.3-Env (Fig. 3A). A comparison of mean Env-mediated HIV transfer calculated for each participant showed that Env isolated from VNPs or RPs display similar functional features (Fig. 3B).

The binding of Env to CD4 is known to trigger intracellular signals leading to a stabilization of acetylated α-tubulin^5^. To evaluate potential differences in signaling activity between VNP- or RP-Envs, pseudotyped viruses bearing selected Env were incubated with CEM.NKR-CCR5 cells and the level of viral infection and related α-tubulin acetylation were assayed. Hence, HIV-1 luciferase-reporter pseudoviruses were produced using a pNL4.3-Luc-R-E- vector and each of the different R5-tropic *env* expression clones from VNP and RP individuals, and an R5-tropic reference BaL strain. This technical approach, using the same viral backbone with different *envs*, allowed the generation of viral particles to infect the same permissive cells for the analysis of the functional involvement of each viral Env in signaling and early infection. Equal amounts of luciferase reporter pseudoviruses were used to infect CEM.NKR-CCR5 cells providing a quantification of HIV-1 infection in the absence of other viral factors and of any influence of late steps of viral infection^5,21,22^. We observed a heterogeneous efficiency for early infection in Envs isolated from either VNP or RP individuals (Fig. 3C) resulting in similar mean infectivity for participants from both groups (Fig. 3D). A similar scenario was observed when α-tubulin acetylation, a key Env/CD4-mediated signal for productive fusion and infection, as previously described^5,23^, was analyzed in CEM.NKR-CCR5 cells. We observed heterogeneous ability to trigger acetylation of α-tubulin (Fig. 3E, Supplementary Figs 1 and 3F) by the different Envs. Although VNPs tended to trigger lower acetylation, the difference of mean values for each participant was not statistically significant (Fig. 3G) Taken together, these data suggest that Envs from VNPs or RPs showed similar CD4 binding affinity and signaling capacity.

### Cytopathicity of Selected Env. Autophagy induction during cell-to-cell contacts

**Figure 4:**
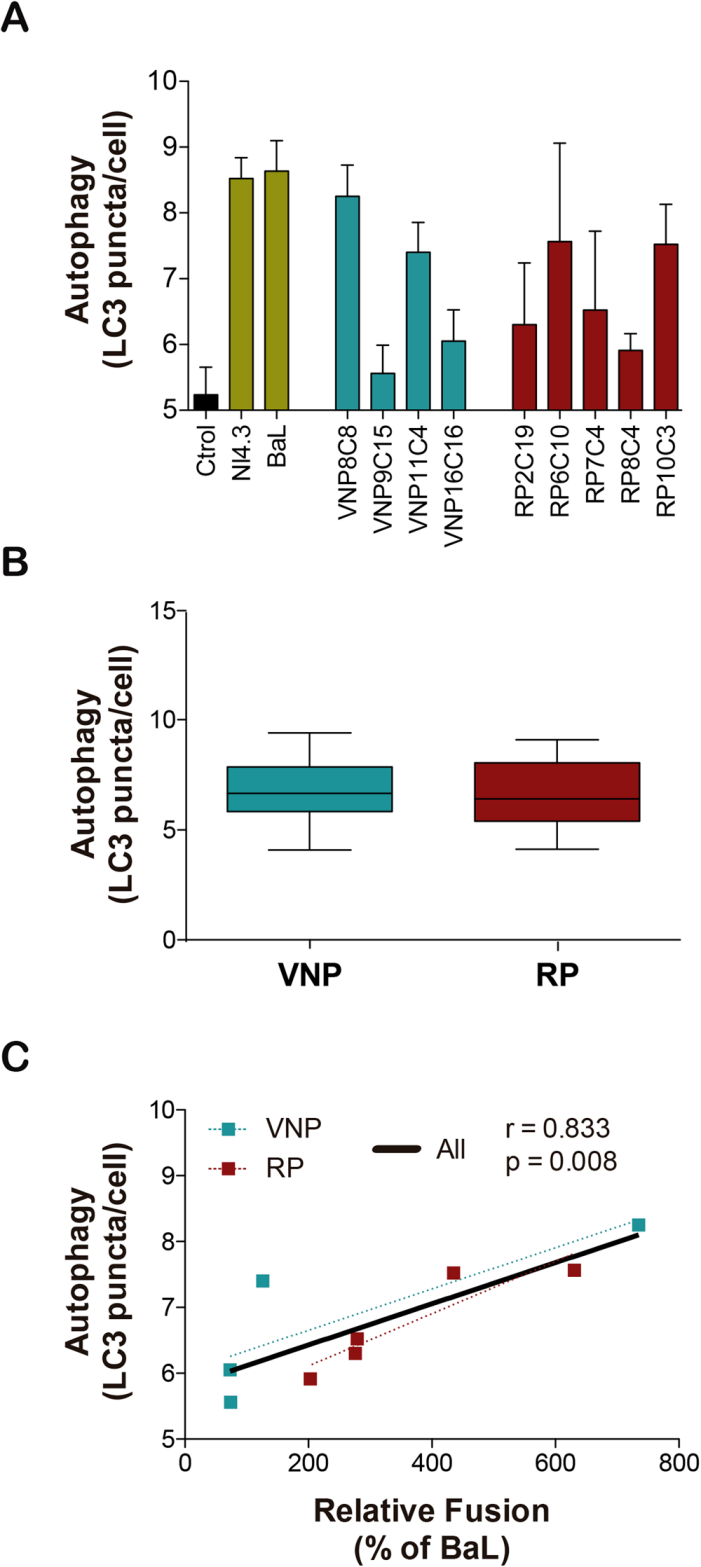
Analysis of the capability of selected HIV-1 Envs isolated from VNP and RP individuals to trigger autophagy. The ability of Env to induce autophagy in uninfected cells was evaluated by co-culturing HEK-293T expressing the indicated Env clones with HUT78 cells. After two days, autophagy was assessed by fluorescence microscopy calculating the number of LC3 puncta per cell. Panel A shows mean ± SEM of four different experiments for the indicated clones. Panel B shows comparison of median values between VNP and RP (green and red, respectively). Panel C shows the spearman correlation of autophagy induction and fusogenic activity for the Env evaluated. Green and red symbols correspond to VNP and RP clones respectively. Linear regression is also shown for illustrative purposes (black line corresponds to all data, blue and red dotted lines correspond to VNP and RP data, respectively).

Finally, we evaluated the ability of the selected Envs to induce autophagy in uninfected CD4^+^ T cells. Indeed, autophagy is triggered in uninfected cells after their contact with infected cells expressing Env, leading to cell death^24–26^. However, all *in vitro* models of autophagy induction have involved X4- and R5-tropic Envs from laboratory-adapted HIV-1 strains (NL4.3 and BaL, respectively). We thus evaluated the ability of primary R5 Envs, isolated from VNP and RP individuals, to trigger autophagy in CCR5^+^ T cells. Co-culture of the CCR5^+^/CD4^+^ HUT78 T-cell line with HEK-293T cells expressing primary Envs triggered the autophagy pathway in bystander uninfected CD4^+^ T cells, as measured by the number of LC3 puncta per cell after 2 days of co-culture. Both NL4.3- or BaL-expressing cells were used as positive controls (Fig. 4A). Although no significant difference between both groups was noticed (Fig. 4B), the level autophagy induction strongly correlated with the fusogenic activity of Env (Fig. 4C). These observations confirm the link between fusogenic function and autophagy, thereby suggesting that no differences in Env-mediated cytopathicity are expected among VNPs and RPs.

## Discussion

Understanding viral factors associated with the VNP phenotype in HIV-1 infection may help to unveil cytopathic mechanisms of CD4^+^ T cell depletion *in vivo*. However, most approaches addressing potential virological and immunological determinants of the VNP phenotype have failed to identify a well-defined candidate. Here we evaluated the role of Env in the maintenance of sustained CD4^+^ T-cell counts in VNP individuals. Env is a key determinant of HIV-1 cytopathicity and its fusogenic activity has been shown to be a key determinant in CD4^+^ T cell loss, both *in vitro* and *in vivo*^16,27,28^. Moreover, defective specific Env functions, such as CD4 binding and signaling, have been recently identified in a group of LTNPs showing undetectable viremia and slow progression to AIDS^5^.

To address the role of Env in the VNP phenotype, we have analyzed a wide range of Env features. First, the sequence diversity was analyzed to evaluate their impact on functionality. Recently, the accumulation of genetic polymorphisms associated with impaired viral fitness has been described in viruses from isolated VNP individuals^15^. Despite this observation, viral population diversity remains higher in VNPs compared to standard progressors or RPs^15^. Consistently, we found significant higher *env* gene diversity in sequences isolated from VNPs compared to RPs. However, the mean diversity of *env* sequences isolated from each individual did not correlate with their mean fusogenicity. Furthermore, in a single individual, the genetic distance to the patient consensus sequence of each clone was unrelated to its fusogenic capacity. Long-lasting coexistence of HIV-1 replication with host immune responses in VNPs is expected to lead to a continuous Env escape from neutralizing antibodies and accumulation of mutations to compensate cost in viral fitness^29,30^, while in RPs viral replication occurs in severely immunocompromised individuals favoring uncontrolled HIV-1 replication and selection of the fittest variants, as described for late AIDS phase of chronic infection^31^. In summary, our data suggest that Env plasticity allows for the maintenance of proper fusogenic function in both VNP and RP individuals despite representing almost opposite situations of HIV-1 replication.

Several authors have described that Env showing similar fusogenicity may differ in other relevant functions such as cell death induction^16,32^. Therefore, we in-depth characterized different Env functions in our clones, first focusing on the binding of gp120 to CD4. The interaction of Env and CD4 governs the formation of virological synapses^20^, which are involved in HIV-1 spread and in pathogenic events. Moreover, we have recently described a heritable default in CD4 binding and signaling as one of the mechanisms explaining the lack of progression in a cohort of Spanish LTNPs^5^. In contrast to these recent data, the binding of native Env to CD4 showed comparable efficiency in Envs isolated from VNPs and RPs, as measured by the efficiency of virus binding to primary CD4^+^ T cells in the context of cell-to-cell transmission. Analogously, in a cell-free virus infection model, viruses bearing Envs isolated from VNPs or RPs showed similar infectivity and similar ability to promote CD4-dependent signals (as measured by α-tubulin acetylation) in permissive CEM.NKR-CCR5 cells, ruling out an active role of a defective interaction between Env and CD4 in the VNP phenotype.

The involvement of Env in cell-to-cell HIV-1 spread is also relevant for cytopathicity. Indeed, cell-to-cell HIV-1 transmission is able to activate different cell death mechanisms, classically associated with apoptosis, and more recently, with autophagy and pyroptosis^25,33,34^. All of them resulting in the rapid destruction of target cells before being productively infected (bystander cell death)^27,32^. In particular, Env triggers autophagy after cellular contacts between Env expressing and target cells^25^. Autophagy occurs after gp41 activation of membrane fusion^35^, as mutations abrogating fusogenicity abolish both autophagy and cell death^24^. Despite the widely-described role of Env in viral cytopathicity, our data show that Env isolated from VNPs or RPs similarly trigger autophagy in uninfected cells. To our knowledge, this is the first report describing the ability of primary R5-tropic Envs to induce autophagy in uninfected CD4^+^ T cells. Remarkably, although the extent of autophagy induction was independent of the HIV-1^+^ group (VNP or RP), it strongly correlated with the fusogenic capacity of Env, suggesting that autophagy induction promoted by primary isolates follows the same mechanisms described for laboratory adapted Env in T cells^24^. The bystander effect induced by Env has been associated to specific genetic signatures in residues 425 and 476 of gp120^32^ and in residues in the HR1 sequence of gp41^16^. Consistent with the similar cytopathicity of Envs isolated from VNPs and RPs, no sequence differences in those residues were observed between both groups. Similarly, the N362 residue that has been associated with fusogenicity was equally distributed among VNP and RP groups^36^.

Our exhaustive analysis of Env function in VNPs has some limitations. Firstly, the analysis is limited to a reduced number of Env that have been carefully selected to maintain both the mean value and the heterogeneity of the original Env collection. Selection may introduce a bias and may also limit statistical power. Secondly, it is difficult to fully recapitulate in *in vitro* assays the functional complexity of HIV-1 Env. However, despite these limitations, our data suggest that Env function is comparable between VNPs and RPs and does not have a major role in the VNP phenotype, at least in the patients analyzed.

Other authors have explored the role of Nef in the VNP phenotype showing, as for Env, no abnormalities in known Nef functions^14^. In contrast, the *in vitro* replication of viruses isolated from VNP patients seems to be impaired as a result of the accumulation of genetic polymorphisms associated with low viral fitness^15^. However, this fact unlikely explains our data, since *in vivo* HIV replication is comparable in VNP and RP groups, as assessed by plasma VL. The lack of a clear role for viral factors in the VNP phenotype may suggest a prominent role for immunological factors. Interestingly, no role for CTL or neutralizing responses have been identified^9,11,37^; nevertheless the VNP phenotype seem to be more prevalent in pediatric HIV-1 infection, suggesting that young immune system is more prone to coexist with high levels of viral replication^10,37^. Furthermore, lower CCR5 expression and lower activation have been found in young non progressors compared to progressors^37^ and long lived central memory CD4^+^ T cells have been identified in pediatric and adult VNPs^12,37^. Such an immunological privilege could be eroded by efficient viral replication of functional viruses present in VNPs and these individuals may eventually develop AIDS when left untreated^38^.

The VNP phenotype in HIV-1 infection could be reminiscent to HIV-2 infection, as in many HIV-2+ individuals, disease progression occurs slowly^39^. *In vitro* studies suggest that HIV-1 and HIV-2 share similar ability to bind to CD4, cell tropism and viral genetic variability^40,41^. Moreover, both HIV-1 and -2 trigger autophagy in permissive infected cells, although virus-mediated autophagic cell-death mainly occurs in HIV-1 infected cells^42^. However, HIV-2 infection is commonly associated to a sustained control of viral replication^43,44^, thus being more similar to the well-defined long-term non-progression associated to natural virological control in HIV-1 infection^39^ than to the VNP phenotype.

In summary, our data demonstrate the lack of functional differences in Env functions (including autophagy induction in bystander cells) among VNP and RP individuals. Exhaustive comparative analysis of the effect of HIV-1 and HIV-2 replication in autophagy pathways and a better knowledge of immunological status of VNPs will be needed to unveil the final virological and/or immunological mechanisms underlying the VNP phenotype.

## Methods

### Samples and full-length Env clones

The isolation of full length Env clones from VNPs and RPs has been previously described^9^. Briefly, VNP individuals without antiretroviral therapy for at least two years (and naive for fusion inhibitor treatment) were identified at the Hospital Germans Trias i Pujol (Badalona, Spain). Criteria for VNP selection were: VL > 10,000 copies RNA/mL and levels of CD4^+^ T cells >400 cells/mL with a loss of CD4^+^ T cells <50 cells/mL/year. For comparative purposes, a matched group of RPs was selected from the Centro Sanitario Sandoval (Hospital Clínico San Carlos, IdISSC, Madrid, CAM). Rapid progression was defined by CD4^+^ T-cell levels <350 cells/mL within 3 years after seroconversion, documented by a HIV negative test within one year before the first positive test. A collection of 100 full-length *env* clones (36 from VNPs and 64 from RPs) were isolated and characterized^9^, and a smaller set of 32 clones (15 from VNPs and 17 from RPs) were further selected for deeper analysis. All full-length *env* sequences were amplified from plasma samples by standard nested PCR and cloned in pCDNA3.1 plasmids as described^9^.

### Cells

The human CEM.NKR-CCR5 HIV-1-permissive cell line (Catalog No. 4376), the HEK-293T cell line (Catalog No. 103), the TZM-bl cell line (Catalog No. 8129) and the HUT78 cell line (Catalog No. 89) were from the NIH AIDS Research and Reference Reagent Program. Cells were cultured and used as previously described^5,22,45^.

### Env expression and fusion assays

HEK-293T cells were co-transfected with the pTat plasmid^46^ and plasmids coding for different Env clones using CalPhos (Clontech). Of note, to avoid transfection variability, all Envs were co-transfected and expressed the same day for the different comparative assays performed. As a negative control, HEK-293T cells were transfected only with pTat. HEK-293T cells were chosen as effector cells since they provide sensitive measures of fusion even when using low fusogenic Env^47^. 24 hours post-transfection, cells were collected, and tested for Env surface expression and fusion activity.

To test Env expression, 2 × 10^5^ Env/Tat co-transfected HEK-293T cells were incubated with 2G12 and IgGb12 monoclonal antibodies (mAbs, Polymun, Viena, Austria) at 4 mg/mL each for 40 minutes at 37 °C. After washing the cells, the PE-labeled goat anti-human IgG (Jackson ImmunoResearch Laboratories) was added and incubated at room temperature for 15 minutes as described^48^. Cells were washed, fixed in formaldehyde 1%, acquired in a FACS LSRII flow cytometer (BD Biosciences) and analyzed using the Flow-Jo software (Tree Star Inc.) The percentage of Env-positive cells and the Mean Fluorescence Intensity (MFI) of these cells were used to evaluate Env expression.

To test fusion activity, 1 × 10^4^ Env/Tat-transfected or control Tat-transfected HEK-293T cells were mixed (ratio 1:1) in 96-well plates with CD4^+^CXCR4^+^CCR5^+^ TZM-bl reporter cells for 6 hours. Luciferase activity was measured (Fluoroskan Accent, Labsystems) using Brite-Lite (PerkinElmer) and normalized to Bal-Env-mediated fusion as described^9,47^. NL4.3- and BaL-Env expression plasmids were used as positive controls for Env staining and as reference value for fusion activity (BaL = 100%).

### Production of pseudoviruses

Replication-deficient luciferase-reporter pseudoviruses were obtained as described^5,21,22^, using the luciferase-expressing reporter virus HIV/*Δnef*/*Δenv*/*luc* (pNL4-3.Luc.R-E- provirus, lacking Env expression and bearing the *luciferase* gene inserted into the *nef* open reading frame; Catalog No. 6070013, NIH AIDS Reagent Program) and the above-described *env* expression plasmids (isolated or reference Env). Viral stocks were normalized by p24 content measured by ELISA (Lenti-X™ p24 Rapid Titer Kit, Clontech, Saint-Germain-en-Laye, France). Alternatively, for infectivity analyses in TZM-bl cells, pseudoviruses were obtained by co-transfecting HEK-293T cells with the HIV-1 backbone-coding plasmid PSG3 (NIH AIDS Research and Reference Reagent Program) and the above-described *env* expression plasmids, isolated or reference Envs^49^. Titers were analyzed using standard methods^50^.

### HIV-1 transfer/CD4 binding

To test viral transfer activity, which exclusively depends on the binding of gp120 to the CD4 molecule^5^, Env expression plasmids were co-transfected with the Env-defective pSG3 plasmid in HEK-293T cells. One day after transfection, 1 × 10^5^ HEK-293T cells were mixed at a 1:1 ratio in 96-well plates with primary CD4^+^ T cells freshly isolated from healthy donors by negative selection (CD4^+^ T-Cell Isolation Kit II, human, Miltenyi Biotec)^19,20^. Viral transfer was assessed after 2 hours of incubation by staining cells with the anti-HIV-1 p24 Kc57 mAb (Coulter) as described^45,51^. Cells were acquired in a FACS LSRII flow cytometer and the content of p24 in gated CD4^+^ T cells and gated HEK-293T cells was analyzed using the Flow-Jo software (Tree Star Inc.). The percentage of p24^+^ HEK-293T cells was used as a control for transfection efficiency and was similar among all experiments. Since coreceptor binding or fusion activity are not required for viral transfer, the frequency of p24^+^/CD4^+^ T cells was a direct measure of the amounts of HIV-1 virions bound to or taken up by target cells.

### Luciferase viral infection assay

CEM.NKR-CCR5 cells (9 × 10^5^ cells in 24-well plates containing RPMI supplemented with 20 μg/mL of Polybrene) were infected with 500 ng of p24 of luciferase-reporter pseudoviruses, bearing VNP- or RP-Envs, in 1 mL total volume with RPMI 1640 for 2 hours (by centrifugation at 1,200 rpm at 25 °C) and subsequent incubation for 4 hours at 37 °C, as previously described^5,21,22^. As a control for R5-tropic viral infection, a BaL.01-*env* plasmid (catalog number 11445, NIH AIDS Research and Reference Reagent Program) was used. Unbound virus was then removed by washing the infected cells. After 48 hours of infection, luciferase activity was measured using a luciferase assay kit (Biotium, Hayward, CA) with a microplate reader (VictorTM X5; PerkinElmer, Waltham, MA). When indicated, permissive cells were pretreated with an anti-CD4 neutralizing mAb (5 μg/mL; eBioscience, San Diego, CA).

### Signaling activity assays

For signaling activity assays, western blot analyses of HIV-1 Env-mediated α-tubulin acetylation were performed in CEM.NKR-CCR5 cells (1 × 10^6^ cells) incubated with 500 ng of p24 of luciferase-reporter pseudoviruses for 90 minutes at 37 °C^5,23^, bearing VNP- or RP Envs. As indicated above, the R5-tropic BaL.01-*env* was used as a positive control. Membranes were first probed with anti-acetylated α-tubulin 6-11B-1 mAb, then gently stripped (30 *minutes at RT with stripping buffer: Glicine 7,5* *g, SDS 0,5* *g (w/v), Tween-20 1% (v/v), pH 2,2 in 500* *mL water; then 5* *minutes at RT with washing buffer: NaCl 150* *mM; and again membrane balance in Tris buffered saline Tween-20 buffer*) and blotted with the anti-α-tubulin B-5-1-2 mAb (both from Sigma-Aldrich St. Louis, USA), and secondary antibodies conjugated with horseradish peroxidase (HRP, Dako, Glostrup, Denmark) were used to detect key bands on blots. The level of α-tubulin acetylation was quantified and expressed as the ratio of the intensities of the acetylated α-tubulin to the total α-tubulin bands, as described^5,23^.

### Autophagy assays

The ability of Env clones to induce autophagy in CD4^+^ T cells was assessed by co-culturing for 2 days HEK-293T cells transiently expressing Env (effector cells) with the target HUT78 T-cell line. Cell-surface Env expression was analyzed as described above. Autophagy was analyzed by immunofluorescence after endogenous LC3 staining. Cells were fixed in a 1:1 acetone/methanol mixture for 5 minutes at −20 °C. Cells were washed in PBS and incubated with the rabbit anti-LC3 antibody L7543 (1 µg/mL, Sigma Aldrich) for 1 h, washed, and then incubated with Alexa Fluor® 488 goat anti-rabbit IgG (2000 × diluted, Life Technologies) for 30 minutes. After washing, nuclei were labeled with DAPI and cells were examined by epifluorescence using a Leica microscope as described^35^. The number of LC3 dots (green) per CD4^+^ T cell was counted. More than 100 CD4^+^ T cells were analyzed by 2 independent investigators.

### Genotypic analyses

Full-length Env clones were sequenced using Big DyeTM Terminator Cycle in an ABI Prism 3730 XL Sequencer (Applied Biosystem, Life Technologies) in the Genomic Unit of the CNM-ISCIII. Sequences were assembled using the SeqMan program (DNASTAR Inc, Madison, WI). The program Bioedit Sequence Aligment Editor (version 7.1.3.0) was used to align and manually edited the nucleotide sequences obtained. A mean of 8 clones per patient were sequenced. Maximum likelihood trees were constructed and phylogenetic confidence was assessed by bootstrap analysis of 1,000 replicates using MEGA (Version 6.0) program. To estimate viral heterogeneity, we determined the mean pairwise nucleotide distances, and the standard error, between all sequences obtained for each patient. The genetic distance of each clone to the patient consensus sequence was also estimated by the best-fit model of nucleotide substitution.

### Statistics

Statistical analyses were performed using GraphPad Prism, version 5.0b (GraphPad Software) and R package version 3.5.2. Significance when comparing groups was determined with a 2-tailed nonparametric Mann Whitney U test or by non-parametric Kruskal-Wallis test with Dunn’s correction for multiple comparisons. Non-parametric Spearman test was used to calculate correlations. Association between parameters was analyzed taking into account repeated measures from different Env of each individual by fitting linear mixed effect models with random intercepts. The proportion of variance explained by the fixed predictors (R^2^) was calculated using the Nakagawa and Schielzeth’s approach^52^.

### Statement

We confirm that all methods were carried out in accordance with relevant guidelines and regulations. All procedures followed the Helsinki Declaration in 1975, as revised in 1983, and were approved by the Ethics committee of the Hospital Germans Trias I Pujol. All individuals provided their written informed consent.

## Supplementary information

Supplementary Figure 1
