# Supplementary material for: HIV-1 envelope glycoproteins isolated from Viremic Non-Progressor individuals are fully functional and cytopathic
Source: Sci Rep. 2019 Apr 3;9:5544. doi: 10.1038/s41598-019-42075-3 (PMC6447548; doi:10.1038/s41598-019-42075-3)
Supplement: Supplementary file 1 — Supplementary Figure 1 [file 41598_2019_42075_MOESM1_ESM.pdf]

Title page for Supplementary Figure 1

HIV-1 envelope glycoproteins isolated from Viremic Non-Progressor individuals are fully functional and cytopathic

Romina Cabrera-Rodríguez[1], Veronique Hebmann[2], Silvia Marfil[3], María Pernas[4], Sara Marrero-Hernández[1], Cecilia Cabrera[3], Victor Urrea[3], Concepción Casado[4], Isabel Olivares[4], Daniel Márquez-Arce[1], Silvia Pérez-Yanes[1], Judith Estévez-Herrera[1], Bonaventura Clotet[3,5], Lucile Espert[2], Cecilio López-Galíndez[4], Martine Biard-Piechaczyk[2], Agustín Valenzuela-Fernández[1,*] & Julià Blanco[3, 5,*]

[1]Laboratorio de Inmunología Celular y Viral, Unidad Virología y Microbiología del IUETSPC, Unidad de Farmacología, Sección de Medicina, Facultad de Medicina, Universidad de La Laguna (ULL), La Laguna 38071, Tenerife, Spain.

[2]Institut de Recherche en Infectologie de Montpellier (IRIM), Université de Montpellier, CNRS, 34293 Montpellier, France.

[3]AIDS Research Institute IrsiCaixa, Institut de Recerca en Ciències de la Salut Germans Trias i Pujol (IGTP), 08916 Badalona, Barcelona, Catalonia, Spain.

[4] Unidad de Virología Molecular. LRIR. Centro Nacional de Microbiología (CNM), Instituto de Salud Carlos III, 28220 Majadahonda, Madrid, Spain,

[5]Universitat de Vic-Central de Catalunya, UVIC-UCC, Vic 08500, Catalonia, Spain

* **Correspondence** should be addressed to J.B. (jblanco@irsicaixa.es) and A.V-F. (avalenzu@ull.edu.es).

Cabrera-Rodríguez *et al.*
Supplementary Figure 1

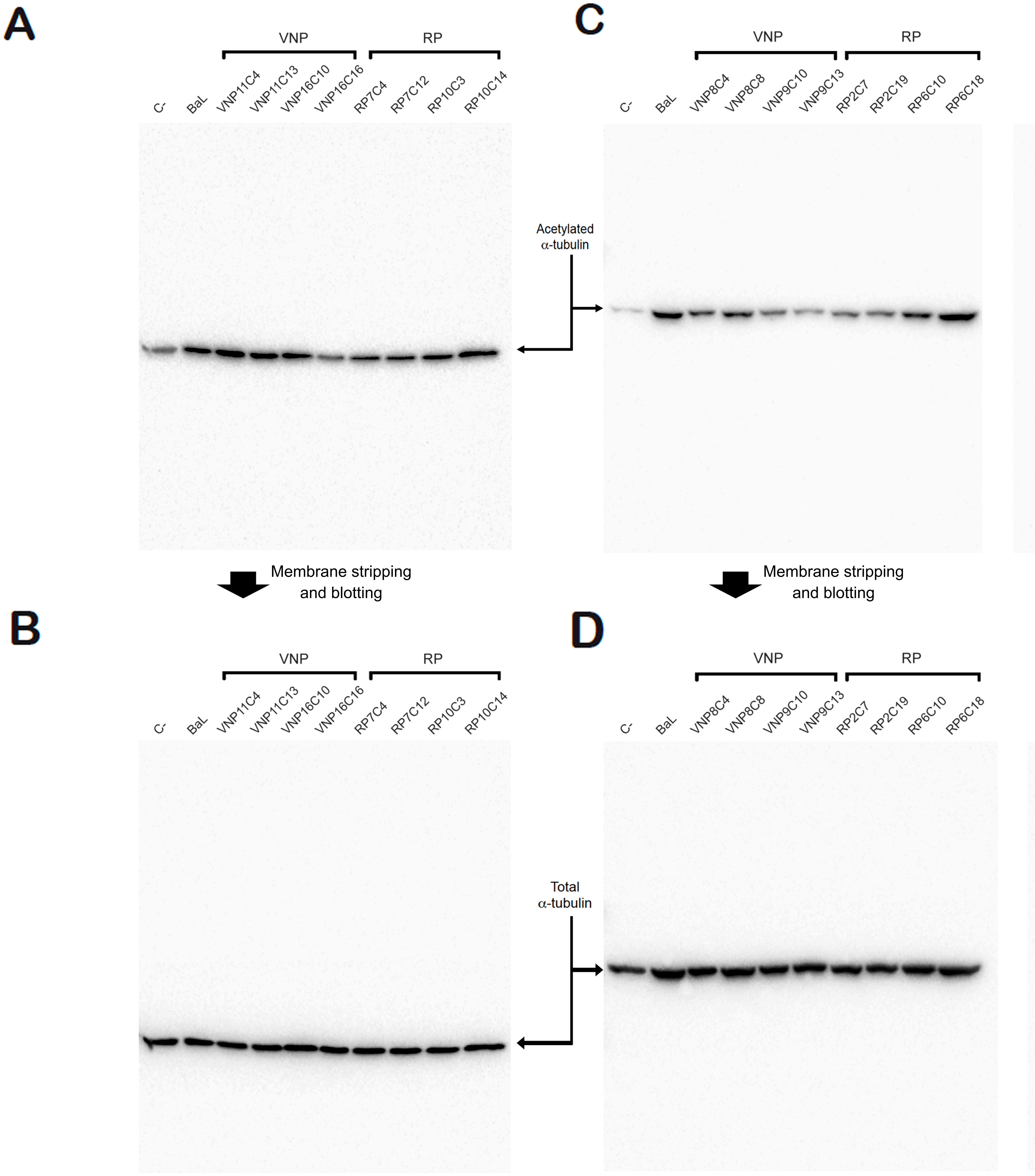

Legend to Supplementary Figure 1.
Analysis of HIV-1 Env-mediated CD4 mediated signaling induced by selected primary Envs isolated from VNP and RP individuals: *full-length gels/blots of data represented in Fig. 3E.*
Panels **A** and **B** show full-length gels/blots for acetylated α-tubulin and total α-tubulin labeling, respectively, and under the indicated experimental conditions. In fact, these two panels show the same gel/blot that was firstly blotted with an anti-acetylated α-tubulin Ab (panel **A**), then gently stripped, and further blotted with an Ab against total α-tubulin (panel **B**), as indicated in Methods section. Key bands in these two blots are represented in main Fig. 3E, left blots. In Panels **C** and **D**, it was similarly proceeded with this gel/blot, and the key bands from these two blots are represented in main Fig. 3E, right blots.
